# EXERGAMES IN ADOLESCENTS: ASSOCIATED FACTORS AND POSSIBLE REDUCTION IN SEDENTARY TIME

**DOI:** 10.1590/1984-0462/;2019;37;4;00019

**Published:** 2019-10-10

**Authors:** Iazana Garcia Custódio, Adriano Akira Ferreira Hino, Cristiano Copetti Rodriguez, Edina Maria de Camargo, Rodrigo Siqueira Reis

**Affiliations:** a Universidade Tecnológica Federal do Paraná, Curitiba, PR, Brazil.; b Pontifícia Universidade Católica do Paraná, Curitiba, PR, Brazil.; c Universidade Federal do Paraná, Curitiba, PR, Brazil.; d Washington University in St. Louis, Brown School, Saint Louis, Missouri, United States of America.

**Keywords:** Motor activity, Video games, Adolescent behavior, Atividade motora, Jogos de vídeo, Comportamento do adolescente

## Abstract

**Objective:**

To describe the use of exergames, associated factors and to quantify the time attributed to the use of exergames within the time spent on video games in a sample of adolescents from Curitiba, Paraná, Brazil.

**Methods:**

This was a cross-sectional study that evaluated frequency and weekly volume of physical activities using the Physical Activity Questionnaire for Adolescents. Weekly frequency and daily time of use of exergames and videogames were self-reported. Mann-Whitney and Kruskal Wallis tests were used to compare the time spent playing exergames, and Poisson regression was used to test the associations (p<0.05).

**Results:**

495 adolescents were interviewed (51.3% girls), predominantly aged between 12 and 13 years (41.3%), under/normal weight (60.4%), medium socioeconomic status (39.8 %) and from public schools (69.3%). Most of the participants did not have video games in their bedroom (74.3%) and did not reach recommended levels of physical activity (55.5%). One in five adolescents used exergames (16.4%). Age (RP: 0.54; 95%CI 0.30-0.97, p=0.039) and having a console in the bedroom (RP: 1.89; 95%CI 1.27- 2.81, p=0.002) were associated with exergame use. Male sex (X_: 195.0; AIQ: 486.3; p=0.024) practice of leisure time physical activity (X_: 160.0; AIQ: 350.0; p=0.048) were associated with weekly volume of exergame use.

**Conclusions:**

Overall, less than two out of ten adolescents used exergames, and the use was higher among young adolescents and those who had a console in their bedrooms. Volume of use was higher among boys and those performing more than five hours of leisure time physical activity per week. In addition, a considerable part of the time devoted to the use of video games, was in fact, destined to the use of exergames.

## RESUMO

**Objetivo:**

Descrever o uso de *exergames*, os fatores associados e quantificar o tempo atribuído ao uso de *exergames* dentro do tempo total de uso de *videogames* em uma amostra de adolescentes de Curitiba, Paraná.

**Métodos:**

Estudo com delineamento transversal no qual a frequência e o volume semanal de atividades físicas foram avaliados com o Questionário de Atividade Física para Adolescentes. A frequência semanal e o tempo diário de uso de *exergames* e de *videogames* foram autorreportados. Os testes U de Mann-Whitney e Kruskal-Wallis compararam o tempo de uso dos *exergames*; e a regressão de Poisson, as associações (p<0,05).

**Resultados:**

Foram entrevistados 495 adolescentes (51,3% meninas), predominantemente com idade entre 12 e 13 anos (41,3%), baixo peso/normal (60,4%), nível socioeconômico médio (39,8%) e de escolas públicas (69,3%). A maioria não possuía *videogame* no quarto (74,3%) e não cumpria a recomendação de atividade física (55,5%). Um em cada cinco adolescentes usava *exergames* (16,4%)*.* A idade (razão de prevalência [RP] 0,54; intervalo de confiança de 95% [IC95%] 0,30-0,97; p=0,039) e a posse de *videogames* no quarto (RP 1,89; IC95% 1,27-2,81; p=0,002) foram associadas com o uso. Ainda, o sexo masculino (X_: 195,0; AIQ: 486,3; p=0,024) e a prática de atividade física no lazer (X_: 160,0; AIQ: 350,0; p=0,048) apresentaram associação com o volume semanal.

**Conclusões:**

Ao todo, menos de dois em cada dez adolescentes utilizam *exergames*, sendo a frequência maior entre os mais novos e que possuem consoles de jogos nos quartos. O volume de uso é maior entre os meninos e entre adolescentes que praticam mais do que cinco horas de atividades físicas de lazer na semana. Além disso, parte considerável do tempo destinado ao uso de *videogames* foi, na realidade, destinada ao uso de *exergames*.

## INTRODUCTION

Several factors have contributed to the reduction of physical exercise among young people. Among these factors are the growing process of urbanization, the reduction of public spaces for physical activity, the increase in violence, the technological dependence (when the individual cannot control their own use of the internet / games / cell phones) and the various facilities gained from modernization.^1^ ^,^ ^2^ In view of these changes, there is a transition from predominantly outdoor physical activities to those performed indoors, which appear to be safer, but may lead to a more sedentary lifestyle.^3^ ^,^ ^4^ Data presented by the National School Health Survey (PeNSE) indicate that more than half of ninth graders spend three hours or more on an average weekday on sedentary activities such as watching television, using a computer, playing video games or doing other activities while sitting down.^5^

In this context, the growing interest in video games in the adolescent population is perceived as one of the leisure activities in this age group.^6^ However, while traditional video games are classified as sedentary activity, ^6^ ^,^ ^7^ ^,^ ^8^ active or exergames stimulate movement through motion sensors^.^ ^9^ ^.^ ^10^ This feature has made exergames one of the alternatives for increasing physical activity levels among young people, ^11^ in addition to their potential for developing cognitive, motor and spatial orientation skills.^12^ ^,^ ^13^ ^,^ ^14^

Despite the increased use of exergames, most of the questionnaires for assessing sedentary behavior only measure screen time, which may include the time adolescents spend on exergames.^15^ ^,^ ^16^ This approach may result in misclassification, and may overestimate the time spent on sedentary activities if the time in exergames is not discriminated. A study on young Australians, found that 42% of teenagers who play video games also use exergames. The authors found that 20% of the total time adolescents reported playing video games was spent playing exergames, indicating that approximately one fifth of sedentary behavior in relation to time, was being misclassified.^17^

Although there are already a considerable number of studies on the use of exergames,^11^ ^,^ ^18^ most studies have aimed to evaluate the effects of exergames on several outcomes.^19^ ^,^ ^20^ Still, descriptive studies on the use of this new technology are, in their entirety, from countries with a different socioeconomic context, which may not represent the Brazilian reality. Thus, investigating this new alternative of physical activity may represent a breakthrough in studies on sedentary behavior, since few studies have explored this possibility. Therefore, the objective of this study was to describe the use of exergames and the factors associated with their use, as well as to verify the time spent playing exergames within the total time of video game use in a sample of adolescents from Curitiba, Paraná.

## METHOD

The data used in this study is part of the International Physical Activity and Environment Network (IPEN) project, a multicenter study conducted in 19 countries. The study has a cross-sectional design, conducted with a household survey and face-to-face interviews. All participants - guardians and adolescents - signed an informed consent form, and the study was approved by the Research Ethics Committee of Pontifícia Universidade Católica do Paraná(PUC-PR) (135-945 / 2012). Sampling was performed in multiple stages. Firstly, 32 census tracts within the city of Curitiba were collected, and each sector contained 15 adolescents. The 2,395 census tracts of the city were classified according to income and walkability characteristics. As an income indicator, the average household income was used, according to the Brazilian Institute of Geography and Statistics (IBGE) in 2010.

Walkability is the English term that seeks to identify areas with favorable design and characteristics for everyday walking. In the present study, this concept was operationally composed of an indicator, considering mixed land use, street connectivity, and residential and commercial densities. The sector income and walkability indicators were classified into deciles. Next, the second and third deciles were selected as those with low income and walkability, while the census tracts located in the eighth and ninth deciles were determined as those with high income and walkability. Thus, census tracts were selected from the four quadrants resulting from the combination of income extremes and walkability. Finally, eight census tracts from each quadrant were selected: high income and low walkability; low income and low walkability; low income and high walkability; and high income and high walkability, totaling 32 census tracts.^22^ The quadrants contained in these sectors were considered and the listing process was performed in all quadrants and households. The researchers addressed all households within the sector and, when they found a household where residents met the eligibility criteria, they invited them to participate in the project.

The study included adolescents between 12 and 17 years of age. Adolescents who lived less than a year in the neighborhood or who had any physical and / or cognitive limitations that prevented the practice of physical activity were not considered eligible. The final study sample consisted of 495 adolescents. The calculation of the power performed *a posteriori*, considering an alpha of 0.05, an association strength of 1.7 and a prevalence of outcome of 16%, allows the identification of associations with a power of 0.75.

Twenty-three interviewers, including undergraduate and postgraduate students, conducted face-to-face interviews after receiving 12-hour theoretical-practical training on how to conduct the approach at home and with participants, selection criteria, conduction of interviews, filling out forms and identifying the refusal rate. Data collection was performed between August 2013 and May 2014.

Physical activity performed by adolescents was assessed using the Adolescent Physical Activity Questionnaire (QAFA).^23^ This questionnaire was first developed in checklist format for North American adolescents, ^24^ which was translated and adapted for Brazilian adolescents.^23^ The questionnaire consists of a list of 24 moderate to vigorous physical activities, with the possibility for adolescents to add activities beyond those listed. The questionnaire showed good reproducibility (ICC = 0.88; 95% CI 0.84-0.91) and concurrent validity when compared with a 24-hour recall (r = 0.62; p <0.001).^23^ The level of leisure time physical activity was calculated from the sum of the weekly time in each of the listed activities. Regarding analysis, the variable was categorized as “up to 419 minutes per week” and “420 minutes per week or more” according to the physical activity recommendations for adolescents.^25^ ^,^ ^26^

The use of exergames was assessed by the question “Consider the activities you perform outside of school. In a normal week, do you play active games (Xbox, Wii, etc.)? ”, The possible answers were“ Yes ”or“ No”. If the answer was yes, the weekly frequency of use (days / week) and the daily duration (minutes / day) were also questioned. Through these two variables it was possible to calculate the weekly volume of use (minutes / week).

Video game ownership was assessed with the following question: “Indicate if you have these items in your room. - Video games (Xbox, Playstation, Nintendo Wii)”, with “Yes” and “No” being the possible answers. Video game usage time was also measured with the question “How long do you play video games on your device or computer on a normal school day?”, With the possible answers being “None”, “15 minutes / day” , “30 minutes / day”, “1 hour / day”, “2 hours / day”, “3 hours / day” and “4 hours or more / day”.

The adolescent’s sex was recorded by the interviewer and the age was obtained based on the date of birth and the date of the interview, and classified into three age groups (“12-13 years”, “14-15 years” and “16-17 years”). Body mass and height were measured using a digital scale and a stadiometer which was used to calculate body mass index (BMI), and then categorized into four levels (“underweight”; “normal weight”; “overweight”). ”and “obesity”)^27^. Socioeconomic status (NSE) was assessed using a standardized questionnaire based on ownership of property at home, education of the financial head of the household, and the presence of domestic workers.^28^ For analysis purposes, the NSE was classified into three levels: “high” (classes A1 + A2), “medium” (classes B1 + B2) and “low” (classes C1 + C2 + D + E). The schooling of the guardian was put into three levels: “Complete elementary school”, “Complete high school” and “Complete superior level education”. The adolescents reported the type of school they attended (private or public), formal work (yes or no) and grade repetition / year (never, once or twice, 3 or more).

Sample characteristics were described by absolute and relative frequency distribution. The normality of variables with continuous measurement scale (time of use of exergames) was verified using the Kolmogorov-Smirnov test. The data did not present normal distribution, thus, for description, we used, in addition to the mean and standard deviation, the median and interquartile range. The Mann-Whitney and Kruskal-Wallis U-tests were performed in order to compare the duration of use of exergames, according to sociodemographic variables, possession of video games in the bedroom and the level of leisure time physical activity. The comparison between the proportions was performed using the chi-square test. Poisson regression was used to test the association between the use of exergames and sociodemographic variables, the fact of having video games in the bedroom and the level of leisure time physical activity. Descriptive statistics measured the time spent playing video games and how much of this time was attributed to exergames. All analyzes were performed using SPSS 20.0 software, and the significance level was maintained at 5%.

## RESULTS

Among the 495 adolescents (51.3% girls) included in the final sample (Table 1), there was a higher proportion of adolescents aged 12 and 13 years (41.3%), nutritional status classified as underweight / normal weight (60.4%).), an average NSE (52.9%) and parents or guardians with complete high school (39.8%). Most respondents studied in a public school (69.3%), never repeated (70.5%), did not work (91.1%) and did not have video games in their room (74.3%).

**Table 1:** Description of demographic characteristics, education and physical activity practice of study participants, Curitiba, Brazil (n = 495).

	Total	
	n	%
Sex		
Male	241	48.7
Female	254	51.3
Age (years)		
12 and 13	204	41.2
14 and 15	167	33.7
16 and 17	124	25.1
BMI		
Underweight+normal weight	299	60.4
Overweight	142	28.7
Obese	54	10.9
SEL 2014		
A1+A2	42	8.5
B1+B2	262	52.9
C1+C2+D+E	191	38.6
Guardian’s schooling		
Illiterate + 4th Grade	84	17.0
Complete primary school	72	14.5
Complete high school	197	39.8
Higher level education complete	142	28.7
School		
Private	152	30.7
Public	343	69.3
Repeated one year		
Never	349	70.5
1 time	100	20.2
2 times or more	46	9.3
Works		
No	451	91.1
Yes	44	8.9
Has a videogames console in bedroom		
No	368	74.3
Yes	127	25.7
Leisure time PA		
Up to 419 min/wk	293	59.2
420 min/wk or more	202	40.8
Uses *exergames*		
No	414	83.6
Yes	81	16.4

BMI: body mass index; SEL: socioeconomic level; PA: physical activity.

Approximately half of the participants (55.5%) practiced some type of leisure time physical activity e (Table 1), and less than two out of five used exergames (16.4%). Among those who used exergames, weekly use was 3.0 ± 2.2 days / week (min .: 1; max: 7; median: 2.0) for 97.5 ± 84.1 minutes / day ( min: 10; max: 420; median: 60.0), with a weekly amount (days / week * minutes / day) of 373.3 ± 542.7 minutes / week (min: 10; max: 2100 median: 120.0).

The use of exergames (Table 2) was only higher for boys (p = 0.024) and for those who did 420 min / wk. or more physical activity during their leisure time (p = 0.048).

**Table 2:** Average values and interquartile ranges according to sociodemographic, health and physical activity variables with time spent using exergames in adolescents from Curitiba, Brazil (n = 81).

	Time spent playing exergames		
	Average	IQR	p-value
Sex			
Male	195.0	486.3	0.024
Female	90.0	240.0	
Age (years)			
12 and 13	203.0	253.9	0.103
14 and 15	166.0	246.9	
16 and 17	122.0	231.7	
BMI			
Underweight+normal weight	120.0	240.0	0.396
Overweight	120.0	360.0	
Obese	180.0	1395.0	
SEL 2014			
A1+A2	160.0	420.0	0.621
B1+B2	120.0	300.0	
C1+C2+D+E	120.0	280.0	
Guardian’s schooling			
Illiterate + 4th Grade	165.0	280.0	0.762
Complete primary school	120.0	727.5	
Complete high school	112.5	300.0	
Complete Higher-level education	155.0	660.0	
School			
Private	210.0	270.0	0.210
Public	120.0	300.0	
Repeated one year			
Never	120.0	300.0	0.542
1 time	105.0	285.0	
2 times or more	225.0	465.0	
Works			
No	120.0	300.0	0.696
Yes	240.0	.	
Has a video game console in the bedroom			
No	120.0	215.0	0.204
Yes	210.0	1095.0	
Leisure time PA			
Up to 419 min/wk.	90.0	191.3	0.048
420 min/wk. or more	160.0	350.0	

IQR: interquartile range; BMI: body mass index; SEL: socioeconomic level; PA: physical activity.

Table 3 presents the bivariate associations on the use of exergames with sociodemographic, health and physical activity variables. Age and video game ownership in the bedroom were the only variables associated with the use of exergames. Adolescents 16 and 17-year-old adolescents old have 46% lower prevalence of using exergames compared to 12-and 13-year- old adolescents (p = 0.039). Those who had video games in the bedroom had 89% more prevalence of use when compared to those who did not (p = 0.002).

**Table 3:** Bivariate associations between sociodemographic, health and physical activity variables with the use of exergames (n = 81).

	n	%	Plays exergames		
			PR	95%CI	p-value
Sex					
Male	42	17.3	1		
Female	39	15.1	0.88	(0.59-1.31)	0.533
Age (years)					
12 and 13	40	19.5	1		
14 and 15	28	16.7	0.85	(0.55-1.32)	0.486
16 and 17	13	10.6	0.54	(0.30- 0.97)	0.039
BMI					
Underweight+normal weight	47	15.5	1		
Overweight	25	17.4	1.12	(0.72-1.74)	0.615
Obese	9	16.7	1.06	(0.55-2.03)	0.860
SEL 2014					
A1+A2	11	26.2	1		
B1+B2	43	16.3	0.62	(0.35-1.11)	0.112
C1+C2+D+E	27	14.1	0.54	(0.29-1.00)	0.050
Schooling of the guardian					
Illiterate + 4th Grade	8	9.4	1		
Complete primary school	13	18.1	1.89	(0.83-4.31)	0.127
Complete high school	34	17.1	1.81	(0.87-3.74)	0.109
Complete Higher-level education	26	18.3	1.92	(0.91-4.05)	0.086
School					
Private	25	16.1	1		
Public	56	16.1	0.99	(0.64-1.52)	0.973
Repeated one year					
Never	59	16.7	1		
1 time	16	15.8	0.94	(0.57-1.56)	0.831
2 times or more	6	12.8	0.77	(0.35-1.68)	0.515
Works					
No	78	17.0	1		
Yes	3	6.8	0.39	(0.13-1.19)	0.101
Has a video game console in the bedroom					
No	49	13.1	1		
Yes	32	24.8	1.89	(1.27-2.81)	0.002
Leisure time in PA					
Up to 419 min/wk.	39	15.2	1		
420 min/wk. or more	39	19.1	1.24	(0.83-1.86)	0.283

PR: prevalence ratio; 95%CI: 95% confidence interval; BMI: body mass index; SEL: socioeconomic level; PA: physical activity.

**Graph 1:**
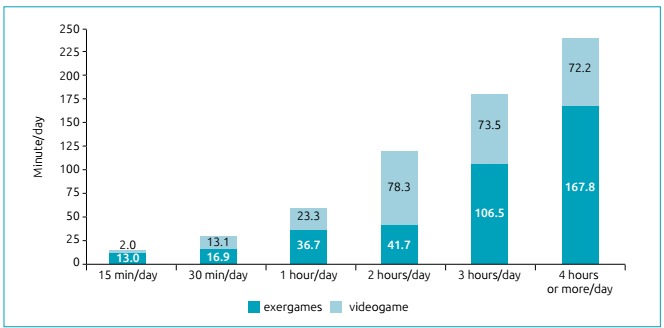
Average time spent on exergames (minutes / day) according to video game use categories among adolescents from Curitiba, Brazil (n = 81).

Graph 1 shows the video game usage time (minutes / day) attributed to the use of exergames (minutes / day). Exergame usage volumes exceeded more than half of total video game usage time in almost all usage categories, with the exception of 2 hours / day.

## DISCUSSION

Understanding the aspects related to the use of exergames and their relationship with the total time of video game use may allow a better understanding of the sedentary behavior of adolescents. The present study identified that less than two out of ten adolescents use exergames, and that the frequency of use is higher among younger adolescents and among those who have video games in their bedroom. Among those who use exergames, the use is approximately five and a half hours per week, and is higher among boys and those who practice more than seven hours of leisure time physical activity. In addition, a considerable part of the time devoted to video games was actually devoted to the use of exergames. Thus, suggesting possible classification errors in the measurement of sedentary time when using global screen time as measurements.

The present study identified that 16.4% of adolescents reported using exergames. In a study^21^ conducted in 2012 in Montreal, among the 1,241 students interviewed, 24% use exergames, i.e. almost a quarter of the total sample. Although these are differently developed countries, the difference between the proportions is 7.6 percentage points, which shows that the proportion found in this study is moving as expected, even though it is not a representative sample study.

Literature reports that the older the adolescents, the lower the use of exergames. O’Loughlin et al.^21^ identified 48.8% use frequency of exergames in the 14-15 age group and 23.0% among adolescents aged between 16 and 17. Similar results were observed in Portugal in 2013,^20^ when the use of exergames was 18.7% for 14-year olds and 16.6% for 16-year olds. The same study reported that 18-year old adolescents were less interested in the practice of exergames. It is speculated that available exergames are unattractive to older teenagers, since most games are dances or sports such as golf, tennis and boxing, and such activities are less common and less preferable for this age group. In addition, typical changes related to this age group such as the start of higher-level education and even entering the labor market, may influence social preferences and habits and thus diminish interest in exergames.

No significant difference in use was identified between genders, however, among the users of exergames, boys used the devices longer than girls. Bailey (2011) ^10^ highlights that different games bring greater satisfaction for both boys and girls, but, in general, boys like them more than girls. It should be mentioned that boys are more physically active than girls, and therefore they may be more interested in exergames.

Among adolescents who reported using video games, about 40 to 80% of the time is directed to the use of exergames. Among those who reported staying up to 1 hour playing video games, approximately 61.2% of the time was spent on exergames. Fullerton et al.^17^ observed that 20% of the time spent on video games was devoted to the use of exergames. In absolute terms, for every hour spent playing video games, 12 minutes would be spent on physical activity rather than sedentary behavior. These results have two important practical implications. Firstly, studies focused on sedentary behavior, specifically screen time, which do not differ from exergames and video games, are overestimating sedentary behavior values. Thus, exergames must be evaluated separately from traditional or non-active video games.

Secondly, the amount of time teenagers spend on using exergames can contribute to reducing sedentary behavior. For example, in the present study, among the adolescents who reported staying 240 minutes / week or more playing traditional video games, 167.8 minutes / week was spent on exergames, which would contribute to a 70% reduction in sedentary behavior. However, this logic would be applicable considering that teenagers replace the screen time of traditional video games with exergames. Thus, future studies should specifically analyze the impact that new technologies are having on adolescents’ habits.

Some limitations should be considered regarding the proper interpretation and extrapolation of results. The sample is not representative of adolescents from the city, since the youths were selected from intentionally selected census tracts to allow for comparisons between walkability and income characteristics of city regions. The selection process considered the spatial and income distribution, which may somehow contribute to the approximation with the characteristics of the city’s adolescents; as well as the use of questionnaires, which have limitations regarding the accuracy of the analyzed activities and possible overestimation in the reported amounts (time spent with video games). However, the questionnaires allowed us to identify the use of video games, exergames and the time dedicated to both, contributing to the understanding of sedentary behavior in adolescents.

The study results have important implications for practice, particularly for investigations related to sedentary behavior using questionnaires. Caution should be exercised in investigating sedentary behavior, as the questionnaires used only capture screen time, which may include the time adolescents spend on exergames - and as seen in the present study, should not be classified as time spent on sedentary behavior. Still, the potential that this type of game can have in relation to physical inactivity should be considered in strategies which promote physical activity.

We conclude that only 16.4% of adolescents in Curitiba use exergames. Frequency of use is higher among younger adolescents who have game consoles in their bedrooms. On the other hand, the volume of use was higher among boys and adolescents who practice more than five hours of leisure-time physical activity per week. Given the considerable proportion of adolescents who use the exergames and the time allocated for this activity, instruments that evaluate screen time as an indicator of sedentary behavior, without distinguishing use of exergames from video games, may be overestimating time spent on sedentary behavior.
